# Mechanistic Insight into the Antioxidant and Antimicrobial Activities of Palm Oil-Derived Biomaterials: Implications for Dental and Therapeutic Applications

**DOI:** 10.3390/ijms26146975

**Published:** 2025-07-20

**Authors:** Syafira Masri, Nurulhuda Mohd, Noor Hayaty Abu Kasim, Masfueh Razali

**Affiliations:** 1 Department of Restorative Dentistry, Faculty of Dentistry, Universiti Kebangsaan Malaysia, Jalan Raja Muda Abdul Aziz, Kuala Lumpur 50300, Malaysia; syafiramasri@ukm.edu.my (S.M.); nurulhuda.mohd@ukm.edu.my (N.M.); 2 Department of Restorative Dentistry, Faculty of Dentistry, Universiti Malaya, Kuala Lumpur 50603, Malaysia; nhayaty@um.edu.my; 3 Mesomorph Worldwide Sdn. Bhd., Kuala Lumpur 52200, Malaysia

**Keywords:** antimicrobial activity, antioxidant properties, palm oil compounds, three-dimensional bioprinting, tissue engineering

## Abstract

Palm oil is a highly versatile natural resource that has gathered significant attention due to its bioactive properties, particularly its antimicrobial and antioxidant effects. Rich in tocotrienols, tocopherols, and carotenoids, palm oil exhibits potent antioxidant activity, while its fatty acid content and other bioactive molecules contribute to its antimicrobial efficacy against various pathogens. The underlying mechanisms of action driving these bioactivities involve intricate molecular interactions, biochemical pathways, and redox processes, which influence microbial cell function and oxidative stress reduction. This review provides a critical analysis of the current mechanistic understanding of palm oil’s biofunctional properties, emphasizing its potential incorporation into engineered biomaterials. Particular focus is given to the chemical composition, reaction pathways, and synergistic potential of palm oil derivatives in material-based formulations. Furthermore, the potential applications of palm oil as a standalone or synergistic agent in novel therapeutic and industrial formulations are explored. By elucidating the mechanistic basis of its bioactivity within material contexts, this review highlights palm oil’s promising role in the development of advanced functional materials for pharmaceutical and dental technologies.

## 1. Introduction

In recent years, the field of dentistry has experienced remarkable progress, especially with the emergence of biomaterials in the realms of tissue engineering and regenerative medicine. Bone regeneration is effectively achieved through the utilization of autogenous bone blocks, which have been recognized as the gold standard in grafting materials [1]. Concurrently, conventional methods employing tissue grafts obtained from living donors or cadavers continue to be extensively applied in dentistry and various medical fields for the restoration of missing or damaged tissues. However, these approaches have limitations, including the potential for infection and the risk of transplant rejection [2]. Moreover, the choice of appropriate treatments, including guided tissue regeneration and bone grafting, is influenced not only by the size and configuration of the osseous defects [3] but also by patient-specific factors such as genetic predisposition and immune response to grafting materials. Therefore, regenerative medicine offers a novel alternative by integrating tissue engineering with the self-healing ability of humans to regenerate, repair, or replace tissues and to restore their impaired function [4,5].

Biomaterials play a pivotal role in regenerative medicine by offering solutions that effectively integrate with native tissues, enhance healing, and restore functionality. Scaffold materials are primarily characterized by their ability to promote cell attachment, stimulate targeted protein production, and facilitate bone tissue development [6]. The typical approach involves introducing cells into a scaffold, which serves as a structural framework to shape the new tissue and deliver signals that support its regeneration [7]. Regenerative biomaterials are distinguished by key biological properties such as biocompatibility, biodegradability, osteoinductivity (the ability to induce osteoprogenitor cells to differentiate into osteoblasts), and osteoconductivity, which refers to providing a scaffold that supports bone cell attachment and new tissue formation [6]. These properties are crucial for inducing cell adhesion, promoting specific extracellular matrix protein synthesis, and supporting the regeneration of bone and dental tissues.

Depending on their origin and composition, biomaterials are broadly classified into three categories: natural, synthetic, and composite materials [8]. Each class offers unique advantages in regenerative procedures. Natural biomaterials, such as palm oil derivatives, are valued for their inherent biocompatibility and ability to deliver bioactive molecules, although they may lack sufficient mechanical strength. In contrast, synthetic biomaterials offer tunable degradation rates and mechanical resilience but often require surface modification to enhance bioactivity [9]. Composite biomaterials combine the strengths of both types, optimizing structural integrity and biological function, making them highly suitable for addressing complex tissue defects.

Natural vegetable oils and fatty acids sourced from edible plants, including palm oil, soybean oil, jojoba oil, rapeseed oil, olive oil, and canola oil, have been extensively studied for their potential in biomaterial polymer formulations. The unique properties of these plant-based components improve the performance, biodegradability, and biocompatibility of biomaterials, enabling more effective and sustainable methods in tissue regeneration treatments [10]. Among them, palm oil is a notable candidate for biomaterial development due to its low molecular weight, ester functional groups, and amorphous nature, which can improve the efficacy of tissue engineering applications [11]. Thus, this review paper will explore the benefits of bioactive components in palm oil and their potential role in advancing biomaterials for regenerative medicine.

Palm oil ranks among the most widely utilized vegetable oils globally, found in approximately 50% of commonly used food and consumer products, including snacks and cosmetics. From 1995 to 2017, the global production of palm oil increased from 15 million tonnes to 66 million tonnes [12]. As of 2020, Malaysia is a prominent producer, with oil palm plantations covering 5.87 million hectares. The nation accounted for 34.3% of the worldwide palm oil market and contributed 18.3% (17.37 million tonnes) to the total global oils and fats sector [13]. In addition to its economic importance, palm oil encompasses bioactive compounds like tocopherols, tocotrienols, carotenoids, and phytosterols, which show potential antimicrobial and anti-inflammatory effects [14].

Palm oil is derived from the fruit of the palm plant (*Elaeis guineensis*) and is one of the most produced and widely consumed edible oils worldwide. The oil palm features a tall, unbranched trunk topped with 40 to 50 pinnate leaves, each approximately 4 to 5 m long. The fruits of the oil palm tree are large, round, and red, growing in clusters of up to 2000, and the oil is extracted mainly from both the pulp and the kernels [15]. The utilization of palm oil as a biomaterial provides a sustainable alternative to the production of bio-based materials. Besides, this approach may also enhance waste production utilization, enabling bioenergy harvesting and new biomaterials development. Consequently, these materials are essential for developing innovative natural polymeric formulations through radiation methods. Notably, they have applications in multiple industrial sectors, including paint resins, nanoparticles, scaffolds, nanocomposites, and lithography [16].

Thus, palm oil fruit phytochemistry has been extensively studied and is known to consist of diverse bioactive compounds. Palmitic- and oleic-rich semisolid fat is the most abundant component of the oil (comprising triglycerides and fatty acids, including palmitic, myristic, stearic, oleic, and linoleic acids) [17]. It is also composed of 30% tocopherols and 70% tocotrienols from a vitamin E fraction, carotenoids, polyphenols, and phytosterols [18]. Palm oil can be divided into two primary components: a liquid oil (65–70%) referred to as palm olein (m.p. 18–20 °C) and a solid fraction (30–35%) known as stearin (m.p. 48–50 °C) [17]. Palm olein undergoes a series of processes, including refining, bleaching, and deodorizing. In contrast, fractionation entails the meticulous crystallization of glycerides at designated temperatures, facilitating the separation of the solid fraction from the residual liquid.

However, the diverse phytochemical characteristics and fractionation capacities of palm oil are determined by the methods of processing and the particular species of oil palm. The distinctions between the palm oil species play a crucial role in the variations observed in oil yield, fatty acid composition, and the presence of bioactive compounds. The African oil palm (*E. guineensis*) exhibits a significantly higher oil yield than the American oil palm (*E. oleifera*). The *E. oleifera* has been adopted for commercial cultivation primarily due to its high oil yield potential. The *E. oleifera* presents a viable option for hybridization with the African oil palm due to its notable characteristics, including resistance to bud rot disease, a slow rate of height increase, and oil that is rich in unsaturated fatty acids, along with substantial levels of carotenes and tocotrienols [15].

In the field of tissue engineering, an optimal scaffold is expected to facilitate the functionality of growing tissue throughout the regeneration process and to undergo degradation once the tissue has reached maturity [19]. Hydrogels consist of three-dimensional (3D) networks formed by synthetic or natural polymeric chains, interconnected through physical or chemical bonds. They possess the ability to absorb, swell, and release significant quantities of trapped water, solvents, or biological fluids without dissolving [20]. Hydrogels possess the capacity to absorb significant quantities of water, reaching up to 99% of their weight, and exhibit a spontaneous response to various stimuli, including temperature, pH, ionic strength, light, and electric and magnetic fields [21,22]. This unique combination of properties renders them particularly valuable for applications in the biomedical field, notably as drug delivery systems.

Generally, the gel matrix network is permeable, allowing for the loading, retention, and eventual release of drugs or active compounds. However, the release rate is dependent upon the diffusion coefficient of the drug or active compounds within the gel network [23]. Synthetic polymers used in biomaterial processing have specific drawbacks, including high toxicity, non-biodegradability, and elevated costs. Conversely, natural polymeric materials are easy to synthesize, economical, biodegradable, biocompatible, non-immunogenic, non-toxic, and physiologically safe. Thus, the exploration of current uses of palm oil products within the pharmaceutical sector is fascinating as this edible vegetable oil possesses remarkable physicochemical characteristics, including the capacity to alter chemical composition for the advancement of biomaterials [16].

Moreover, the incorporation of natural bioactive compounds into hydrogels has been investigated to improve their therapeutic functionality by providing additional biological benefits. The addition of palm oil facilitates the formation of urethane bonds, which influences the development of hard segments. This enhancement leads to improvements in both Young’s modulus and elongation properties while achieving favorable shape fixity and shape recovery ratios [24,25,26]. Apart from that, palm oil has garnered interest for its ability to reduce oxidative stress, owing to its abundant antioxidant constituents, such as tocotrienols, tocopherols, and carotenoids [27]. Oxidative stress, defined as an imbalance between reactive oxygen species (ROS) and the antioxidant defense system, significantly contributes to chronic diseases [28]. The bioactive compounds in palm oil, especially tocotrienols, demonstrate significant free radical-scavenging capabilities that assist in neutralizing reactive oxygen species and reducing cellular damage [29].

Oxidative stress refers to an imbalance between oxidants and antioxidants that favors oxidants, resulting in disrupted redox signaling and control, as well as potential molecular damage. The fundamental concept is that, throughout an open metabolic system, a steady-state redox balance remains intact at a specific setpoint, establishing a basal redox tone [30]. A deviation from this steady-state redox balance is regarded as a stressor, prompting a stress response. The definition of oxidative stress encompasses two fundamental concepts: (i) a shift in the opposite direction is identified as “reductive stress,” and (ii) physiological deviations that remain within a controlled range are classified as “oxidative eustress,” whereas excessive and harmful deviations are known as “oxidative distress” [31]. The defense against harmful oxidant levels involves various antioxidant enzymes and their supporting systems, along with low molecular mass antioxidants, collectively forming an antioxidant network [31]. Furthermore, recent studies have reported that palm oil exhibits significant protective effects on enamel surfaces when subjected to both erosive and erosive+abrasive challenges, likely due to its interaction with the acquired enamel pellicle and its antioxidant properties [32].

The importance of redox balance in cellular function and tissue repair has led to increased interest in biomaterials that can reduce oxidative stress in engineered microenvironments. The incorporation of palm oil-derived compounds into hydrogels offers a promising strategy for the modulation of oxidative stress in engineered tissues. The strategic incorporation of these compounds into biodegradable polymer matrices in hydrogel may offer localized protection against ROS, which generally hinder cellular function and tissue regeneration.

### 1.1. Oxidative Stress in Bone

Oxidative stress significantly influences bone metabolism, impacting both bone formation and resorption by disrupting the balance between osteoblasts and osteoclasts. Bone defects can arise from various circumstances, including trauma, congenital factors, or disease, affecting millions worldwide [33]. Oxidative metabolism generates ROS as byproducts of energy-producing reactions predominantly occurring in the mitochondria [34]. Reduced levels of ROS can function as signaling molecules essential for regulating cell differentiation, self-renewal, and proliferation. Conversely, elevated levels of ROS are damaging due to their frequent interactions with molecules like proteins, ribonucleic acid (RNA), and deoxyribonucleic acid (DNA), leading to the suppression of the osteogenic lineage. Maintaining bone homeostasis is a critical factor in bone regeneration. During normal bone homeostasis, the differentiation of osteoblasts is promoted through signaling pathways such as fibroblast growth factor (FGF), bone morphogenetic protein, and hedgehog. Concurrently, the differentiation of osteoclasts is regulated by macrophage colony-stimulating factor (M-CSF) and receptor activator of nuclear factor kappa-B ligand (RANKL) [35].

Osteoblasts and osteoclasts are essential in bone remodeling. Hence, they have been studied to enhance understanding of the bone regeneration process. ROS are essential in the regulation of differentiation in osteoclasts. The excessive production of osteoclasts induced by local inflammation was presumed to be preventable by restricting the overproduction of intracellular ROS [34]. Maintaining bone homeostasis is essential for achieving an optimal balance between formation and resorption, which affects bone mass and strength. ROS induce apoptosis in osteoblasts and osteocytes, which are cells located in the bone matrix and derived from mature osteoblasts, thereby promoting osteoclastogenesis [36].

Apart from that, ROS induce a range of responses, including proliferation, growth, differentiation arrest, and cell death, through the activation of various signaling pathways. Mitogen-activated protein kinases (MAPKs), including extracellular signal-regulated kinases (ERK1/2), c-Jun N-terminal kinase (JNK), and p38 MAPK, play a role in the apoptosis of osteoblasts and osteocytes [37]. Elevated levels of ROS inhibit osteoblast activity and differentiation, consequently impairing mineralization and osteogenesis. These events enhance bone remodeling turnover, resulting in alterations and a reduction in bone mass.

Antioxidants have opposing effects, facilitating the differentiation of osteoblasts and promoting bone formation, thereby sustaining essential osteocytes that enhance osteoblast activity and osteogenesis, while simultaneously inhibiting osteoclast differentiation and activity [37]. Several factors produced primarily by osteoblasts and osteocytes regulate the activity of osteoclasts and osteoblasts, thereby influencing bone remodeling. Among these, the most significant are the receptor activator of NF-κB ligand (RANKL) and osteoprotegerin (OPG) [38]. Their expression is responsive to elevated oxidative status, which leads to the upregulation of RANKL and downregulation of OPG via the activation of protein kinases (such as ERK1/2, JNK, etc.) and/or other factors that influence specific transcription factors. RANKL promotes the differentiation and activities of osteoclasts through interactions with specific receptors on preosteoclasts, facilitating osteoclastogenesis and bone resorption.

**Figure 1:**
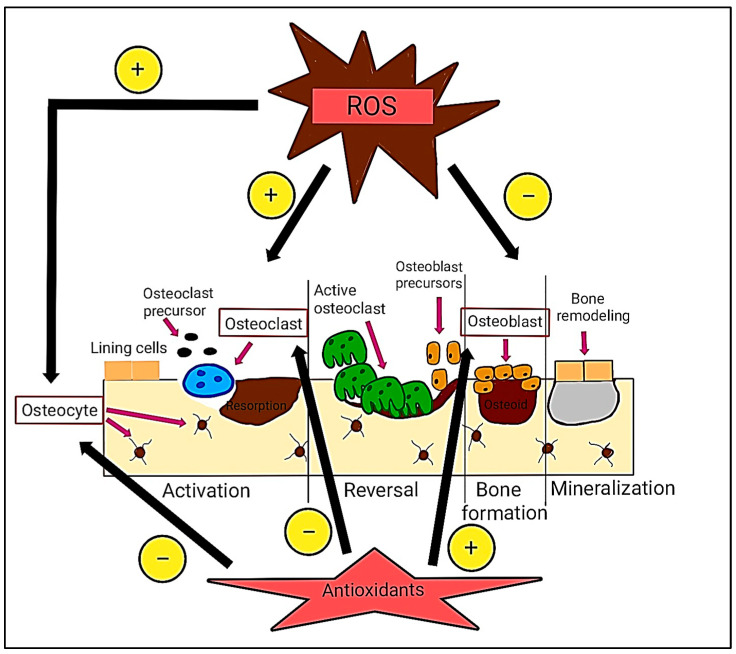
Impact of ROS and antioxidants on bone remodeling. ROS promote osteoclast development and osteocyte death while suppressing osteoblast activity, resulting in enhanced bone resorption. Antioxidants stimulate osteoblast development, inhibit osteoclast activity, and prevent osteocyte death, thus fostering bone growth. Symbols: “+” indicates induction/promotion; “–” indicates reduction/suppression.

Conversely, OPG, which is produced via the activation of the Wnt/β-catenin signaling pathway, acts as a soluble receptor that binds to and inhibits RANKL, thereby reducing osteoclast activity [39,40]. Oxidative stress impedes osteoblast activation and diminishes OPG synthesis. Consequently, RANKL predominates, enhancing osteoclast activity and promoting bone resorption. This results in an elevated bone remodeling rate, as indicated by an increased RANKL/OPG ratio, signifying enhanced bone resorption. The RANKL/OPG ratio regulates the equilibrium between bone resorption and production. A higher ratio indicates more bone resorption without sufficient new bone formation, resulting in increased bone turnover. This imbalance is associated with skeletal disorders such as osteoporosis and bone degradation resulting from inflammation [40,41]. Figure 1 below summarizes the impact of ROS and antioxidants on bone remodeling, illustrating the different functions of ROS in promoting bone resorption and antioxidants in facilitating bone formation.

### 1.2. Oxidative Stress in Dental

In dentistry, saliva serves as the primary defense against free radicals through various antioxidant mechanisms, including glutamate, ascorbic acid, uric acid, and melatonin, as well as antioxidant enzymes such as superoxide dismutase, catalase, and glutathione peroxidase [42]. These components reduce the adverse effects of reactive oxygen and nitrogen species when present in excess in the oral cavity, exceeding the levels required for normal physiological function [43]. Moreover, saliva has been found to be a method for detecting oxidative stress, as these indicators reflect changes in the oral cavity as well as the pH balance and antioxidant capacity in the oral cavity [44]. It is recognized as a fluid with a significant ability to identify molecules that serve as biomarkers for various oral diseases, including periodontitis and dental caries [45]. In addition to saliva, the oral epithelium also serves as a critical barrier, providing mechanical protection and immunological defense against pathogens and oxidative damage, primarily through the action of antimicrobial peptides [46,47].

Oxidative stress significantly contributes to the pathophysiology of various oral diseases, especially periodontal and dental tissue health. Periodontitis is a chronic inflammatory disease that leads to the destruction of the tooth-supporting tissues, including gingival connective tissue, periodontal ligament, and alveolar bone [48]. This tissue breakdown exposes the tooth roots to the oral environment, allowing bacterial biofilms to form and calcify into dental calculus. The disease typically progresses slowly and, if untreated, results in tooth mobility, functional impairment, aesthetic issues, and eventually tooth loss [48]. The development of periodontitis is significantly linked to the overproduction of ROS, mainly by hyperactive neutrophils. When antioxidant defense mechanisms are insufficient to address oxidative imbalance, ROS lead to cellular damage, worsening tissue degradation, and disease severity [49]. Although the exact role of oxidative stress in periodontal breakdown is unknown, there is growing evidence for compromised antioxidant capacity in periodontal tissues and fluids, independent of smoking, and elevated advanced glycation end-products levels both in persons with type 2 diabetes and in smokers, known risk factors for periodontitis [50].

**Figure 2:**
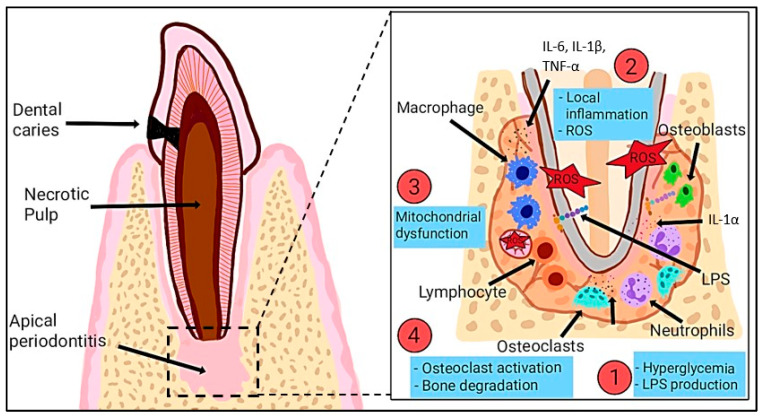
Endodontic infection, such as *E. faecalis* and lipopolysaccharides (LPSs), induces inflammation, drawing polymorphonuclear neutrophils (PMNs) to the gingival sulcus. This results in excessive ROS formation, leading to oxidative stress that harms fibroblasts, triggers osteoblast apoptosis, activates osteoclasts, and degrades proteoglycans, hyaluronan, and collagen, thereby compromising tissue integrity and impeding wound healing.

Furthermore, many studies indicate that palm oil provides protection against enamel erosion and damage caused by abrasion [51,52]. The protective effect is associated with its elevated tocotrienol content, which offers antioxidant advantages, enhances dentine integrity, and promotes dentinal bridge formation [53]. The carotenes present in palm oil may provide cellular protection, thereby improving its significance in oral health. Additionally, its antioxidant and anti-inflammatory properties could potentially help mitigate the harmful effects of dental biofilms and reduce the risk of gingival and pulpal inflammation. Besides, the presence of oral microorganisms within dental biofilms contributes significantly to common oral health issues. Thus, gingival inflammation arises from the interaction between host responses and biofilm microorganisms, influenced by both local and systemic factors [54]. Figure 2 below illustrates the role of ROS in endodontic infections, highlighting how pathogens such as *Enterococcus faecalis* and lipopolysaccharides (LPSs) induce inflammation, resulting in increased ROS production that negatively impacts cells, degrades extracellular matrix components, and delays tissue healing.

## 2. Synthesis and Extraction of Palm Raw Materials

In order to improve productivity and maintain consistent quality, various mechanical and chemical techniques are utilized in the extraction process of palm bioactive compounds. Crude palm oil is extracted using traditional techniques, such as mechanical pressing, sterilizing, and softening of oil palm fruits [55]. In contrast, ultrasonic-assisted extraction (UAE) is an advanced technique that improves extraction efficiency while maintaining the integrity of bioactive substances. In addition to these methods, other extraction techniques such as polycondensation, ethanolic extraction, maceration, reflux, boiling, triturating, and methanolic extraction demonstrate the versatility of oil palm raw materials and their derivatives. Each method yields unique outcomes, enabling the tailoring of extraction procedures to achieve specific goals, especially in the development of value-added palm oil products and the synthesis of bioactive compounds. The combination of different techniques for extraction not only improves the quantity and effectiveness but also expands the potential uses of palm oil across several industrial and biological domains.

### 2.1. Epoxidized Palm Olein

Epoxidized palm olein (EPO) represents a chemically altered version of palm olein, the liquid component derived from palm oil following the fractionation process. Epoxidation is a simple but highly efficient chemical modification of polyolefins that introduces a new reactive group, enhancing characteristics and enabling diverse uses. The high reactivity of the oxirane ring in EPO has made it a promising reactive intermediate, as the epoxy group can be converted into other functional groups through the degradation process. EPO was synthesized into palm oil-based polyester polyols (PPPs) via polycondensation with glutaric acid [56]. The result was a liquid with advantages such as low viscosity and particular molecular characteristics, suitable for industrial applications like foams, adhesives, and coatings [56].

Apart from that, epoxidation can be performed to achieve various conversions, particularly when the product is intended for use in synthetic chemicals. Conversely, epoxidation may be utilized to achieve a product with optimal efficiency, focusing on attaining the highest conversion rate, although not exclusively focusing on maximizing conversion. Previous studies indicate that the in situ peracid epoxidation of vegetable oils, along with the subsequent ring-opening reactions, requires substantially different reaction conditions. Epoxidation is generally performed at moderate temperatures ranging from 40 to 60 °C, employing sulfuric acid as the catalyst [57,58]. In contrast, another study identified that the ring-opening process generally occurs at higher temperatures, i.e., above 80 °C [59]. The observed contrasting conditions arise from the distinct mechanistic requirements inherent to each step of the reaction.

In terms of visual analysis, oleic acid appears as a crystal-clear liquid. However, following the epoxidation process, the epoxidized oleic acid transforms into a white semi-solid or slurry at room temperature [59]. The transformation of palm oil derivatives into more complex, functional materials presents significant potential for their application in sustainable, high-performance products. This is particularly important in the realm of sustainability, as the versatility of palm oil as a renewable resource has the potential to diminish dependence on petroleum-based materials. Briefly, the epoxidation of palm olein in this investigation was conducted utilizing performic acid produced in situ through the reaction of formic acid (HCOOH), serving as the oxygen carrier, and hydrogen peroxide (H_2_O_2_), acting as the oxygen donor [60].

**Figure 3:**
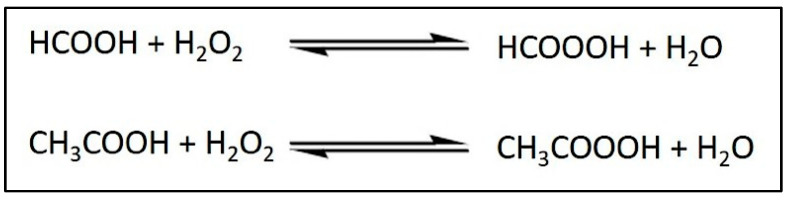
The formation of performic and peracetic acids through the in situ interaction of carboxylic acids and hydrogen peroxide.

The in situ synthesis of performic acid (HCOOOH) and peracetic acid (CH_3_COOOH) as epoxidizing agents was necessary due to the highly exothermic and unstable characteristics of the reaction. The formation of performic and peracetic acids takes place via the in situ interaction of carboxylic acids with hydrogen peroxide, as shown in Figure 3. The epoxidation of palm olein occurs via an electrophilic addition mechanism. In this process, the unsaturated bonds within the triacylglycerol (TAG) structure are transformed into oxirane (epoxide) rings, yielding EPO. Peroxy acids are frequently utilized in these reactions because of their electrophilic oxygen, located between the carbonyl group and the acidic hydrogen. The mechanism encompasses a systematic sequence of bond-breaking and bond-forming processes, during which proton transfer takes place, ultimately resulting in the formation of the epoxide and a carboxylic acid byproduct. This transition state distinctly demonstrates the dynamic exchange of electrons that occurs during the epoxidation process.

### 2.2. Oil Palm Leaves

The oil palm leaves (OPLs) are considered underutilized when compared to other oil palm biomasses. Nevertheless, the low-value OPLs may be utilized for applications in the pharmaceutical sector, given their significant potential as antioxidants, antimicrobials, and disinfectants, particularly in the context of wound healing [61,62]. OPLs contain an abundance of phytochemicals, including flavonoids, polyphenols, carotenoids, and terpenoids, which demonstrate a variety of biological activities. However, there is no universal extraction system, and specifically, no acid hydrolysis procedure that is suitable for the extraction and hydrolysis of all plant constituents, due to the significant diversity of constituents in plants. Consequently, it is imperative to optimize the extraction and acid hydrolysis processes in order to conduct a precise evaluation of phytocompounds from a variety of plant matrices.

Briefly, the extraction method previously documented involved the processing of oil palm leaves through freeze-drying, then grinding them into a fine powder for extraction purposes [63]. In this approach, absolute ethanol was employed at a 1:20 (*w*/*v*) ratio over a duration of 48 h, focusing on its significant affinity for polyphenolic compounds. Chloroform fractionation was utilized to enhance the purity of the extract by eliminating non-polar impurities. The mixture underwent filtration, followed by re-extraction of the residue to improve the recovery of the compound. The final extract went through a drying process to remove any residual solvents.

Besides, the use of ultrasonic-assisted extraction (UAE) on fresh oil palm leaves has demonstrated a significant yield of phenolic compounds, recognized for their antioxidant and anti-inflammatory properties [64]. The optimization conditions, including solvent concentration and extraction time, indicate that ultrasonic methods may significantly enhance the extraction of bioactive compounds while maintaining the integrity of sensitive compounds [64]. Furthermore, the application of maceration and reflux techniques on OPLs has resulted in a yield of 27.26% when using 70% ethanol during the reflux process [65]. This indicates that the method may be beneficial for producing substantial quantities of extract, especially in the context of herbal medicine [65]. Moreover, previous studies showed that UAE decreases phenolic degradation and is more rapid for extracting phenolics from various plants and fruits compared to solid–liquid and microwave-assisted extractions [66,67].

This method may be advantageous for large-scale extraction, especially within the herbal medicine or nutraceutical sectors. The rising global demand for natural health products necessitates the refinement of this method to enhance efficiency and yield, potentially yielding substantial commercial advantages. One challenge is ensuring the purity and stability of the extracted compounds to comply with safety standards for human consumption.

### 2.3. Palm Fruit

Fruits contain various chemical compounds that have significant biological functions. Fruits are recognized as functional foods. The provision of nutrition is essential for the proper functioning of organisms, with their value associated with substances exhibiting protective properties, primarily attributed to their antioxidant activity [68]. Two distinct types of oil are derived from palm fruits: palm oil, which is extracted from the pulp, and palm kernel oil, which is obtained from the kernel. Palm oil is extracted through the mechanical extraction process, specifically by pressing the epicarp and mesocarp of palm fruits. Following the extraction process, the byproduct referred to as palm pressed fiber retains approximately 5–6% residual oil along with a variety of high-value constituents, including carotenoids, tocotrienols, tocopherols, phytosterols, and phenolic compounds [69,70].

The extraction of palm fruit and palm shells using methanol has demonstrated the ability to produce significant bioactive compounds, including tannins, flavonoids, alkaloids, and terpenoids [71]. Boiling and triturating the palm fruit effectively separated the mesocarp from the seed, resulting in the extraction of fatty acids, including hexadecenoic acid and oleic acid, which have various beneficial applications [71]. This method successfully extracted fatty acids from palm fruit. Moreover, the extraction of palm shells using methanol has demonstrated the production of extracts that are abundant in bioactive compounds, notably phenolics and flavonoids, with phenolics exhibiting the highest concentration at 10.4% [72]. This highlights the shells as a valuable, though frequently underutilized, component of the palm tree, with potential applications in pharmaceuticals and various industries [72]. In tissue engineering, antioxidant and antimicrobial functions may have a substantial impact on scaffold design for wound healing, addressing inflammation and contamination from bacteria. The incorporation of bioactive compounds into scaffolds may enhance tissue regenerative capacity and reduce infection risk, thereby improving healing outcomes.

Furthermore, the aqueous extraction of palm fruit has resulted in a 19% yield of extract; however, the lack of comprehensive chemical content analysis highlights the necessity for further research to thoroughly assess its potential [73]. Generally, this extraction technique demonstrates the varied chemical and bioactive potentials of oil palm byproducts. The selection of each method was determined by the specific compounds required, illustrating the potential of customized extraction processes to enhance the yield and purity of target compounds. The findings highlight the adaptability of oil palm-based materials and their potential across multiple industrial, medicinal, and nutritional applications. However, the lack of detailed content analysis for the aqueous extract limits the ability to fully evaluate the potential applications of this method. Thus, further identification is needed to evaluate the potential applications of the aqueous extract in food or cosmetic industries by determining chemical composition. Additional research is required to identify the full range of bioactive compounds that are present in the palm oil byproducts, especially those derived from aqueous extraction, as it emphasizes a potential study gap. Table 1 below presents an overview of the raw materials used, the associated synthesis techniques, and the specific processes involved. Furthermore, it emphasizes the observed results, providing important insights into the efficacy of different methods.

## 3. Bioactive Compound of Palm Oil

Palm oil serves as a substantial source of naturally occurring bioactive compounds, which play a crucial role in its functional and therapeutic properties. Palm oil is rich with natural bioactive compounds such as tocotrienols and tocopherols, which are constituents of the vitamin E family, as well as carotenoids and phenolic acids. Each of these substances demonstrates significant antioxidant, anti-inflammatory, and protective properties. The presence of these compounds enhances the nutritional profile of palm oil and presents promising applications in health, medicine, and biotechnology.

### 3.1. Phenolic Compounds

Phenolics are known as water-soluble compounds that have potent antioxidant activity [77]. Phenolics are hydroxyl derivatives of aromatic carboxylic acids that derive from either benzoic or cinnamic acid groups. The variations in phenolic content can be attributed to factors such as the geographical location of oil palm tree collection, the maturity of the oil palm tree during harvest, the oil palm tree’s growing season, horticultural practices, postharvest storage conditions, and the palm oil extraction preparation method. This is because the phenolic content and antioxidant capacity might decrease as the degree of fruit ripening rises [78].

The most important and the only class of palm oil phenolic compounds comprises the phenolic acids, flavonoids, tannins, and lignans. Phenolic compounds are widespread as secondary metabolites in nature, and they are commonly found in free and bound forms [79]. Flavonoids exhibit high antioxidant activity due to their capability to form highly stable complexes with divalent cations, scavenge free radicals, and terminate chain reactions of radicals. The nucleophilic nature of the aromatic ring in phenolic compounds is more crucial for chelation than the existence of chelating functional groups [80]. Moreover, the antioxidant capacities increase in conjunction with the hydroxylation degree of phenolic compounds. Caffeoylshikimic acid, a significant phenolic compound in the oil palm tree characterized by four hydroxyl groups, could be responsible for its strong antioxidant activity [81].

Flavonoids are plant secondary metabolites, which are responsible for the color and fragrance of flowers [82]. The main subclasses are flavanones, flavan-3-ols, flavanols, isoflavonoids, anthocyanins, and anthocyanidins. Flavonoids have multiple health-promoting activities and are active ingredients in a wide variety of pharmaceutical, nutraceutical, medicinal, and cosmetic formulations. Flavonoids are a large group of naturally occurring phenolic compounds characterized by a C6-C3-C6 three-ring skeleton [83,84]. They are established for their extensive health benefits, largely attributed to their potent antioxidant properties [85]. The two C6 rings, designated A and B, exhibit aromatic characteristics with different degrees of hydroxylation [86]. The C2-C3 bond within the oxygen-containing ring frequently exhibits a C=C double bond structure. The fundamental chemical structure of flavonoids consists of a diphenylpropane skeleton, comprising fifteen carbon atoms in the primary nucleus. This structure includes two six-membered rings connected by a three-carbon unit, which may or may not contribute to a third ring. The metal-chelating properties of flavonoids may contribute to the oxidation of protein residues, either indirectly by generating ROS or through direct electron transfer processes at specific binding sites on proteins [87].

Apart from that, tannins, which are known as polyphenolic compounds present in palm fruit, significantly enhance its antioxidant capacity by neutralizing free radicals and providing protection against oxidative damage. They are anionic natural polymers extracted from the secondary metabolites of plant matrices [86]. This compound is a water-soluble polyphenol with a molecular weight ranging from 500 to 7000 Daltons. Due to its content of carboxyl and hydroxyl groups, tannin can act as a bio-coagulant [88]. Eco-friendly treatment methods utilize coagulants such as anionic and cationic tannins in wastewater treatment. The antioxidant mechanisms of tannins include scavenging of free radicals, chelation of transition metals, and inhibition of pro-oxidative enzymes [89]. Tannins can scavenge radicals like superoxide, peroxyl, and hydroxyl radicals [89]. Procyanidins, which are a class of condensed tannins, show free radical scavenging activity depending on their structural features and degree of polymerization.

### 3.2. Carotenoids

Carotenoids are pigments that produce red, yellow, and orange colours. They are classified as terpenoids, a category of lipids characterized by isoprene as the fundamental structural unit. Among all the genotypes examined, α-carotene and β-carotene were identified as the only predominant carotenoids [90]. Briefly, β-carotene possesses antioxidant properties and serves as a precursor for vitamin A, thus making it a molecule of significant nutritional value [91]. The carotenoid composition in oil palm mesocarp and crude palm oil (CPO) has been reported, with β-carotene recognized as the principal carotenoid, constituting around 55% of the total carotenoids in the mesocarp and 56% in CPO [92]. The increase in β-carotene peak intensity signifies that the fruit is advancing towards maturity. As β-carotene is an orange pigment, the intensity of this pigment increases with the maturity of the fruit [92]. β-carotene is an effective antioxidant that is recognized for its ability to quench singlet oxygen and scavenge peroxyl radicals. However, it is susceptible to degradation from light and heat exposure.

Generally, the antioxidant activity of carotenoids is associated with their role in photoprotection against photo-oxidative damage induced by ROS, which are persistently generated during photosynthesis and aerobic metabolism [93,94]. Photoprotective carotenoids protect cells from oxidative stress through multiple mechanisms, including the dissipation of excess energy as heat via the xanthophyll cycle, scavenging of peroxyl radicals, quenching of singlet oxygen, and inhibition of singlet oxygen formation by deactivating photosensitizers like triplet-state chlorophyll [95,96,97,98]. Carotenoids are categorized into primary carotenoids, which are involved in light harvesting and photoprotection within photosystems, and secondary carotenoids, which serve a photoprotective role but are not associated with photosystems [99]. Primary carotenoids persist in optimal growth conditions, whereas secondary carotenoids are elevated under stress conditions, including excessive light, nutrient deficiency, high salinity, or extreme temperatures, which induce ROS production [100,101].

### 3.3. Tocopherols and Tocotrienols

Vitamin E serves as a significant chain-breaking antioxidant, demonstrating efficacy in reducing oxidative stress and inhibiting the propagation of free radical reactions. Eight naturally occurring forms of vitamin E are identified based on the methylation pattern of the chromanol ring [102]. The compounds are further categorized into four isomers: alpha (α), beta (β), delta (δ), and gamma (γ) tocopherols and tocotrienols [103]. Tocopherols are found in polyunsaturated vegetable oils and the germ of cereal seeds, while tocotrienols are present in the aleurone and subaleurone layers of cereal seeds and in palm oil [104].

Apart from that, tocopherols and tocotrienols share an identical fundamental chemical structure, distinguished by a long chain linked at the second position of a chromanol ring. However, tocotrienols are distinct from tocopherols due to the presence of a farnesyl side chain, in contrast to the saturated isoprenoid C16 side chain found in tocopherols [105]. Both tocopherols and tocotrienols exhibit significant antioxidant properties, particularly in their ability to scavenge lipoperoxyl radicals [106]. A recent finding indicates that tocotrienol is not classified within the vitamin E family due to its unique biological activities, cellular targets, and molecular mechanisms of action, which differ from those of α-tocopherol [107]. Generally, α-tocopherol, commonly referred to as vitamin E, is recognized for its function in preventing ataxia with vitamin E deficiency (AVED) [108]. Additionally, α-tocopherol inhibits protein kinases, cellular activity, cell proliferation, and gene expression. Palm oil concentrate exhibited the highest tocopherol content among lipid components in animal feed, measuring 2318 mg/100 g, with 90% comprising tocotrienols [109]. This figure was 93 times greater than the plant-based fat “Planta,” which had a concentration of 25.05 mg/100 g in frying fat [109].

## 4. Protective Roles of Palm Oil and Its Bioactive Compounds

Palm oil has antioxidant and antimicrobial properties, making it a viable bioactive element for incorporation into scaffolds. Briefly, the antioxidant potential is attributed to tocotrienols, tocopherols, and carotenoids, which neutralize ROS to reduce oxidative stress and preserve cellular components from damage. The antimicrobial properties of palm oil are largely due to its abundant free fatty acids, monoglycerides, and polyphenols, which compromise microbial cell membranes, disrupt metabolic functions, and prevent biofilm development. Thus, understanding these mechanisms is essential for enhancing palm oil-derived biomaterials in tissue engineering applications.

### 4.1. Mechanism of Action: Antioxidant Properties of Palm Oil

Briefly, the antioxidant activity differs between crude and refined palm oils, mainly due to the presence of phenolic compounds [110]. Phenolic compounds in crude palm oil (CPO) significantly contribute to its antioxidant activity. However, the refining process reduces the phenolic content of the oil, resulting in a decrease in antioxidant potential. These phenolic compounds function as hydrogen donors, neutralizing free radicals such as 2,2-diphenyl-1-picrylhydrazyl (DPPH) and chelating metals like iron and copper, thereby inhibiting the formation of detrimental hydroxyl radicals. CPO exhibited significantly greater antioxidant activity than refined oils, such as refined palm oil, reaching a 70% inhibition in DPPH assays. The refining process, which includes bleaching and evaporation, significantly reduces the concentration of bioactive phenolic compounds, thereby accounting for the reduced antioxidant activity observed in refined oils. A lower concentration of phenolic compounds may result in reduced antioxidant capacities [111]. The antioxidant properties of phenolics are attributed to their functions as reducing agents, hydrogen donors, and free radical quenchers [112]. Additionally, phenolics serve as metal chelators, inhibiting the catalytic role of metals in radical initiation processes.

Next, lignin has been identified as a promising natural antioxidant due to its polyphenolic composition. The extraction of lignin from palm oil mesocarp fibers demonstrates significant antioxidant properties [113]. Methanol (MeOH-F) and acetone (ACT-F) lignin fractions exhibited IC_50_ values of 42.5 µg/mL and 43.4 µg/mL, respectively [113]. The results exceeded the findings from commercial antioxidants like BHT (637.7 µg/mL) and Irganox 1010 (1572.7 µg/mL), and were linked to the structural components of lignin, specifically syringyl units and phenolic hydroxyl groups. From this finding, the enhancement of the antioxidant activity of lignin and its fractions can be attributed to their elevated phenolic hydroxyl content, the presence of methoxyl groups, and conjugated double bonds, which collectively stabilize phenoxy radicals. However, the radical scavenging capacity of phenolic compounds is influenced not solely by their potential to generate a phenoxy radical [114]. Groups that stabilize these radicals through resonance enhance the antioxidant capacity of compounds.

Besides, factors such as conjugated carbonyl groups in the side chain, as well as the presence of lignin-carbohydrate complexes (LCC) or fatty acids, adversely influence the antioxidant activity of lignin [115]. In addition, lignin, characterized by lower molecular weight and narrower polydispersity, exhibits better antioxidative effects. The low molecular weight of lignin obtained from lignin-derived products of steam-exploded palm oil lignocellulosic biomass waste exhibited significant radical scavenging activity, approximately 95% for water-soluble lignin (WSL) and 80% for low-molecular-weight lignin (LML), at EC50 values of 0.25 g/L and 0.5 g/L, respectively [116].

Furthermore, palm oil contains beneficial components in small quantities, including carotenoids that contribute to its reddish-orange color and vitamin E, which exists as tocopherols and tocotrienols [117]. To improve the antioxidant capacity, enzymatic treatments employing pectinase and cellulase enhance carotenoid extraction by degrading the cell walls of plant tissues, facilitating the release of carotenoids into the oil. Consequently, it was shown that enzymatic treatments utilizing pectinase and cellulase significantly enhanced antioxidant activity and carotenoid extraction [118]. The combination of both enzymes resulted in a 153% increase in carotenoid extraction. This finding demonstrates the specificity of enzymes in the release of phenolic and carotenoid compounds. Enzymes catalyze the degradation of cell walls, thereby facilitating the release of bioactive components [119]. Thus, the use of pectinase and cellulase enhances antioxidant activity by degrading the cell wall, thereby releasing vital antioxidants into the oil and improving its overall antioxidant capacity.

Besides, tocochromanols and carotenoids, recognized as potent lipid antioxidants, are thought to accumulate in seeds and fruits of plants to protect polyunsaturated fatty acids from peroxidation [90]. It was shown that palm methyl ester (PME) exhibited a higher level of antioxidant activity (69.3%) than crude palm oil (CPO, 30.1%) [120]. Importantly, the PME exhibited a lower IC_50_ value (5.9 µg/mL) compared to CPO (15.6 µg/mL), indicating improved antioxidant efficiency via esterification. Both CPO and PME contain bioactive compounds. However, PME demonstrates superior antioxidant activity attributed to its higher tocotrienol content and the esterification of triglycerides, which improves the extraction and availability of antioxidants [120]. Thus, the PME has a more effective antioxidant capacity than CPO. In this study, the antioxidant properties of palm oil are attributed to carotenoids and tocotrienols, which function as free radical scavengers. In addition, α-carotene and β-carotene, along with lycopene, are vital compounds that function as antioxidants, effectively quenching singlet oxygen.

The antioxidant mechanisms of β-carotene encompass hydrogen transfer, radical trapping, and redox processes. The β-carotene undergoes an addition reaction with peroxyl radicals [90]. The physical quenching of singlet oxygen involves the transfer of excitation energy from singlet oxygen to a carotenoid molecule, resulting in the formation of excited triplet-state β-carotene and ground-state oxygen [121]. The scavenging of peroxyl radicals occurs through the addition of a peroxyl radical to an appropriate double bond in provitamin A or vitamin A molecules, leading to the formation of a carbon radical. This carbon radical can subsequently convert into epoxides or react with additional peroxyl radicals to yield bisperoxyl products [122].

**Figure 4:**
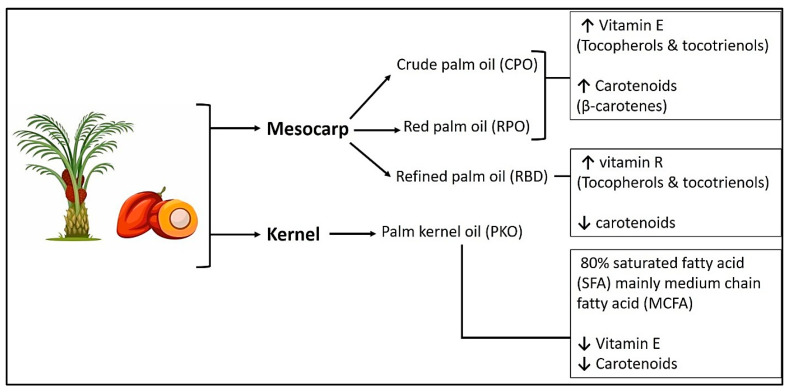
The derivatives of palm oil, derived from the mesocarp and kernel, possess antioxidant and antimicrobial properties, primarily due to the presence of vitamin E (tocopherols and tocotrienols) and carotenoids (β-carotenes).

Furthermore, palm kernel cake has been reported to possess the highest antioxidant activity among palm oil byproducts, primarily as a result of its high phenolic content [79]. Prolonged extraction was observed to enhance phenolic yield. However, it concurrently diminished antioxidant activity as a result of phenolic degradation. Pressed fiber and empty fruit bunches exhibit reduced antioxidant capacities. The extraction process, which includes factors such as duration and solvent ratios, has a substantial impact on the yield of phenolic compounds. However, prolonged extraction times may lead to compound degradation, thereby reducing their antioxidant efficacy. Figure 4 illustrates that palm oil derivatives from both the mesocarp and kernel demonstrate significant antioxidant and antimicrobial properties. The bioactive effects are mainly due to vitamin E compounds, including tocopherols and tocotrienols, along with carotenoids such as β-carotenes. The diverse composition of these natural antioxidants is essential for neutralizing ROS and protecting against oxidative stress, while their antimicrobial properties aid in inhibiting bacterial growth and improving tissue protection.

Moreover, Table 2 below provides a summary of studies exploring the antioxidant properties of palm oil. This table outlines the methods for identifying antioxidant components, the primary bioactive compounds responsible for antioxidant activity, and the mechanisms by which these antioxidants exert their effects. The table presents key findings from each study, providing insights into the potential applications of palm oil as a natural antioxidant in biomedical and therapeutic contexts.

### 4.2. Mechanism of Action: Antimicrobial Properties of Palm Oil

Various components and mechanisms associated with antimicrobial properties offer valuable insights into the potential applications of palm oil and its derivatives in addressing infections, particularly in light of increasing concerns regarding antibiotic resistance. The antibacterial efficacy of palm oil and its derivatives is due to a complex interaction of bioactive components, especially medium-chain fatty acids and natural antioxidants. Bioactive compounds like flavonoids and terpenoids exhibit significant antimicrobial properties, particularly against resistant strains. Compounds like orientin and isoorientin, present in the leaves of palm oil, demonstrate notable antioxidant capabilities and have the potential to work in conjunction with antibiotics to improve their effectiveness [128]. Table 3 below provides an analysis of the antimicrobial activities of various types of palm oil, emphasizing their efficacy against different bacterial and fungal pathogens.

The synergistic antimicrobial effects of red palm oil (RPO) and palm kernel oil (PKO) have been studied in relation to *Propionibacterium acnes* and *Staphylococcus epidermidis* [129]. The effectiveness of PKO is due to its significant concentration of medium-chain fatty acids, especially lauric acid, which compromises bacterial membranes. RPO alone exhibited no antibacterial activity, suggesting that its role is primarily to modify the overall formulation rather than to provide direct antimicrobial effects. The 80:20 PKO:RPO ratio demonstrated notable activity, resulting in inhibition zones ranging from 19.4 to 21 mm for *P. acnes* and from 18.7 to 21.7 mm for *S. epidermidis*. This demonstrates the possibility of optimizing oil ratios to attain specific antimicrobial effects. Moreover, RPO contains a high concentration of carotenoids and tocopherols.

Furthermore, prior research has shown that palm oil contains compounds such as carvacrol, thymol, eugenol, and carotenoids, which have been examined for their antioxidant and antimicrobial properties, rendering them suitable for inclusion in fish gelatin packaging films [130]. The antibacterial effect penetrates bacterial cell membranes, disrupts the lipid bilayer, increases permeability, and causes intracellular leakage. This disruption inhibits essential metabolic processes by interfering with nutrient and ion transport and the inhibition of enzymes [131]. Gram-positive bacteria, including *P. acnes* and *S. epidermidis*, exhibit greater susceptibility due to their simpler cell wall structure relative to gram-negative bacteria, which is responsible for the potent antibacterial activity of PKO.

Furthermore, palm oil derived from the mesocarp has been demonstrated to exhibit significant antibacterial activity against a variety of gram-negative bacteria, such as *Escherichia coli* and *Pseudomonas aeruginosa* [123]. The effectiveness is due to the low pH of palm oil, its high sugar content, and the presence of bioactive compounds such as carotenoids and tocopherols. Delta palm oil demonstrated superior efficacy among various regional variants, particularly against *E. coli*, with inhibition zones ranging from 29.1 to 29.8 mm, exceeding the effectiveness of certain antibiotics. However, the study indicated that heat processing reduced antibacterial activity, presumably due to the degradation of bioactive components. Bioactive compounds such as carotenoids, tocopherols, and free fatty acids destabilize bacterial membranes and disrupt metabolic pathways, resulting in broad-spectrum activity, especially against *E. coli*.

Additionally, the presence of a hydroxyl group (OH-) in phenolic compounds plays a significant role in the killing of bacteria. The interaction of hydroxyl groups with bacterial cell membranes leads to the formation of proton exchangers, resulting in electron delocalization and a reduction in the gradient across the cytoplasmic membrane of bacterial cells [132]. This leads to a destabilization of the proton motive forces and a reduction of the ATP pool in the bacterial cell membrane, ultimately resulting in cell death [133].

Furthermore, the potential of palm oil as an adjuvant in combination antimicrobial treatments is further demonstrated by its ability to improve the efficacy of oxacillin against methicillin-resistant *Staphylococcus aureus* (MRSA) [134]. The proposed mechanism suggests that the fatty acids in palm oil enhance bacterial membrane permeability, thereby facilitating increased antibiotic penetration. The significant synergistic effect, indicated by fractional inhibitory concentration index (FICI) values below 0.5, demonstrates the potential for incorporating palm oil derivatives into treatment protocols for multidrug-resistant pathogens. The synergistic effect likely diminishes bacterial resistance mechanisms, enabling oxacillin to more effectively target intracellular processes, thereby enhancing its bactericidal effect. Lauric acid is classified as a fatty acid. This compound is a saturated medium-chain fatty acid consisting of 12 carbon atoms. Lauric acid is found in substantial quantities (45%) in palm kernel oil, and its elevated levels have been associated with antibacterial properties [135]. It was identified that *S. aureus*, a pathogen identified in the global priority list of antibiotic-resistant bacteria (WHO 2017), exhibited susceptibility to all tested oils, with lauric acid being the most prominent [135]. This finding highlights the potential of palm oil in addressing multidrug-resistant bacteria through combination therapies.

Additionally, a prior study explored the antifungal properties of sophorolipids (SLs) derived from palm oil, with a particular emphasis on pathogens like *Candida albicans* [136]. SLs exhibit amphipathic properties that enable them to penetrate fungal membranes, disrupt biofilm formation, and hinder adhesion and hyphal growth. This property enables SLs to engage with fungal membranes, compromising their integrity through penetration of the lipid bilayer. The disruption of fungal membranes damages their selective permeability, causing the leakage of vital intracellular components and ultimately leading to cell death. Moreover, SLs exhibit antifungal and anti-oomycete activity that is influenced by pH, showing diminished efficacy at elevated pH levels [137]. They effectively inhibit spore germination and mycelial growth of diverse fungal and oomycete pathogens, establishing them as promising agents for antifungal applications. The distinctive antifungal properties of palm oil-derived SLs suggest their potential as effective agents in biomedical applications, especially against fungal infections.

Apart from that, SLs exhibited bactericidal properties when compared to conventional antimicrobial agents, which primarily demonstrate bacteriostatic effects [138]. The research demonstrated variations in emulsification and antifungal effectiveness among strains, with *S. bombicola* exhibiting slightly superior emulsification (60.22%) compared to *S. riodocensis* (54.59%). The antimicrobial effect of SLs on *S. aureus* cells has been previously documented by Díaz De Rienzo et al. (2015) [138]. The cells demonstrated a release of cytoplasmic contents attributed to the intracellular enzyme malate dehydrogenase, suggesting that sophorolipids interact with the cellular membrane, thereby enhancing permeability [139]. In this previous study, a 5% *v*/*v* concentration of SLs (a mixture of congeners) caused disruption of mature maximal biofilms of *B. subtilis* BBK006, as well as in a mixed culture containing both *B. subtilis* BBK006 and *S. aureus*.

In addition, lignin extracted from oil palm empty fruit bunches has been shown to exhibit selective antimicrobial activity [140]. Lignin has demonstrated a significant effect against the gram-positive bacterium *S. aureus*, resulting in nearly complete bacterial reduction within one hour [140]. In contrast, gram-negative *E. coli* exhibited significantly lower susceptibility owing to its complex outer membrane, which impedes lignin penetration. This distinction highlights the structural differences among bacterial types and their influence on antimicrobial effectiveness. Moreover, the present findings align with those of Lee et al. (2014), who investigated the antimicrobial properties of lignin nanoparticles against *S. aureus* and *E. coli* [141]. The lignin nanoparticle sample demonstrated a 99.9% reduction in the population of *S. aureus*. However, it did not impact the population of *E. coli*.

The characterization of lignin as a valuable source of antioxidants, attributed to its diverse array of phenolic compounds and free radical scavenging properties, has enhanced its market value. Lignin possesses antioxidant properties, leading to its diverse applications, including corrosion inhibition, antimicrobial activity, and anti-aging effects [142]. ROS and localized heat produced by light irradiation may be the antibacterial mechanism of lignin breakdown products, disrupting bacterial structures [143]. In addition, the sugar content in lignin may improve its adhesion to bacterial membranes, thereby enhancing its antimicrobial activity [144]. The peptidoglycan layer of bacterial cell walls, containing sugar molecules, interacts with these sugars, enhancing the binding of lignin degradation products [144]. This interaction may enhance antimicrobial activity against *S. aureus*, aiding in the disruption of the bacterial membrane and inhibiting growth. However, major drawbacks of the lignin structure, including its complex architecture, high hydrophobicity, and poor water solubility, have constrained its use in high-value-added applications [145].

Moreover, the antibacterial activity of crude palm oil derivatives, specifically glycerol-fatty acid esters and sucrose-fatty acid esters, has been assessed in previous research [146]. The compounds demonstrated selective activity, inhibiting *S. aureus* while showing no effect on the gram-negative *E. coli*. The resistance of gram-negative bacteria to these compounds is due to their protective outer membrane, which inhibits the penetration of long-chain fatty acids. Generally, palm oil derivatives, including glycerol-fatty acid and sucrose-fatty acid esters, exhibit varying antimicrobial effects influenced by the fatty acid chain length and the specific type of bacterial cell wall. The antimicrobial efficacy of glycerol fatty acid esters, such as monoglycerides and glycerol carbonate esters, was assessed against various bacterial and yeast strains [147]. The research indicates that the antimicrobial properties of these esters are influenced by the structure of the fatty acid chain and the specific type of microorganism involved. The research indicates that short-chain, unsaturated monoglycerides and glycerol carbonate esters featuring cyclic carbonate heads exhibit the highest efficacy as antimicrobial agents, especially against gram-positive bacteria and yeast [147]. The antimicrobial action is thought to involve membrane disruption attributed to surfactant properties.

In addition, a study by Sujarit et al. (2020) discovered that compounds isolated from *Streptomyces palmae* demonstrated antimicrobial properties [148]. The molecules AB204-E and AB204-F exhibited notable activity against gram-positive bacteria; however, they did not show antifungal efficacy. In contrast, anguinomycin A and leptomycin A exhibited broad-spectrum activity against both bacterial and fungal pathogens. This study emphasizes the potential of rhizosphere-associated actinomycetes as a source of novel antimicrobial compounds. The antimicrobial efficacy of compounds derived from *Streptomyces palmae* CMU-AB204T is due to their capacity to disrupt essential microbial processes, including protein synthesis, DNA replication, and membrane integrity. In addition, hydrogels that are produced from palm oil mill effluent have been demonstrated to selectively inhibit the gram-positive bacterium *S. aureus* [149]. Hydrogels such as BDE-MA/PVA (F17) and BCX-MA/PVA (F35) demonstrated significant inhibition zones measuring 31.6 ± 0.85 mm and 30.5 ± 0.60 mm, respectively, but exhibited no activity against *Candida albicans* or *E. coli*. The antimicrobial properties of palm oil mill effluent (POME) hydrogels may involve the disruption of bacterial cell walls or interference with metabolic processes.

**Figure 5:**
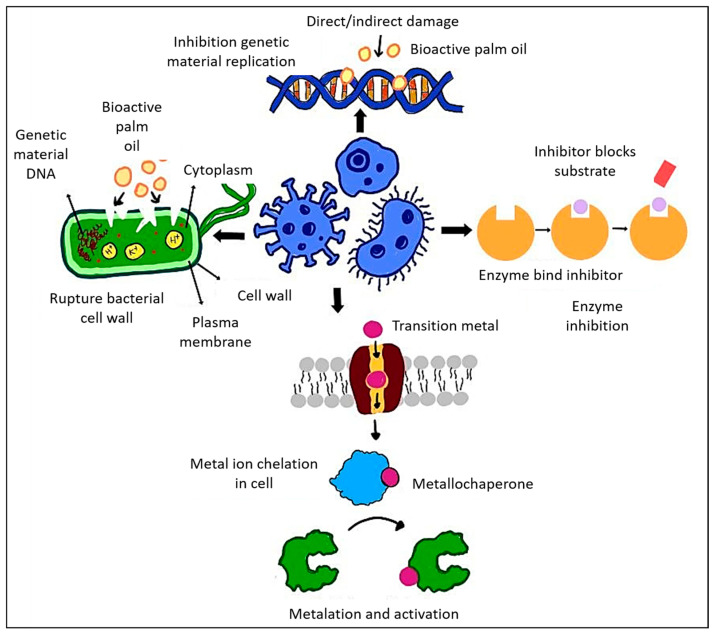
Antimicrobial mechanisms of bioactive palm oil: disruption of bacterial cell walls, inhibition of DNA replication, and modulation of enzyme activity. This illustration demonstrates the antimicrobial properties of bioactive palm oil, emphasizing its capacity to disrupt bacterial cell walls, inhibit the replication of genetic material, and interfere with enzyme function. The chelation of transition metals disrupts metal-dependent processes, thereby compromising bacterial function and survival.

Furthermore, methanolic leaf extracts derived from *E. guineensis* exhibit notable antibacterial and wound-healing properties [150]. In a rat wound model, the extract markedly decreased *S. aureus* bacterial loads, increased fibroblast activity, and facilitated epithelialization and collagen synthesis. On day 16, wounds that received treatment were devoid of bacteria, comparable to those treated with BETADINE^®^, whereas untreated wounds continued to contain bacterial colonies. The methanolic extract derived from the leaves of the palm oil plant possesses bioactive compounds that effectively target and reduce bacterial populations. These compounds likely interfere with the bacterial cell membrane or inhibit essential cellular functions, thereby preventing bacterial proliferation and dissemination in the wound area. Figure 5 below illustrates the antimicrobial mechanisms of bioactive palm oil, highlighting its ability to disrupt bacterial cell walls, inhibit DNA replication, and interfere with enzyme activity. The chelation of transition metals further disrupts essential bacterial processes, leading to decreased bacterial viability. In addition, Table 3 below provides a summary of the antimicrobial properties of various palm oil types against different bacterial species, outlining the bacterial types, mechanisms of action, and significant findings regarding bacterial inhibition and response.

**Table 3:** Antimicrobial mechanisms and findings of different types of palm oil against various bacterial strains.

Type of Palm Oil	Bacteria	Type of Bacteria	Mechanism of Action (Antimicrobial)	Findings	References
Red palm oil (RPO) and palm kernel oil (PKO)	*P. acnes*, *S. epidermidis*	Gram-positive	Lauric acid in PKO disrupts bacterial membranes.	RPO alone had no effect. PKO showed strong activity (up to 23 mm zone); combo (80:20 PKO:RPO) also effective.	[129]
Palm oil from the mesocarp of the oil palm fruit	Clinical and ATCC strains (e.g., *E. coli*, *Pseudomonas*, *Streptococcus*)	Gram-negative	Antimicrobial due to low pH, hydrogen peroxide, and fatty acid content. Heat reduces activity.	Delta palm oil was most effective, especially against *E. coli* (29 mm), better than some antibiotics.	[123]
Palm oil (*Elaeis guineensis*)	*Staphylococcus aureus*, MRSA	Gram-positive	Enhances antibiotic uptake (oxacillin) via membrane disruption.	Palm oil enhanced oxacillin efficacy (FICI < 0.5), showing synergy against multidrug-resistant *S. aureus*.	[134]
Palm oil-derived sophorolipids	*C. albicans*, *S. bombicola*, *S. riodocensis*	Yeast, gram-positive	Amphipathic surfactants disrupt fungal membranes and inhibit adhesion and biofilm formation.	Active against *C. albicans*; surfactants showed emulsification and antifungal potential.	[136]
Oil palm empty fruit bunch	*S. aureus*, *E. coli*	Gram-positive Gram-negative	Lignin disrupts gram-positive cell walls more easily; gram-negatives resist via the outer membrane.	Strong reduction in *S. aureus*; minimal effect on *E. coli*.	[140]
Crude palm oil (CPO)	*S. aureus*, *E. coli*, *P. acnes*, *S. epidermidis*	Gram-positive Gram-negative	Glycerol esters are ineffective; sucrose esters selectively inhibit Gram-positive bacteria.	Sucrose-fatty acid esters inhibited *S. aureus*; others showed minimal antibacterial effect.	[146]
Oil Palm Rhizosphere-Associated Actinomycete, *Streptomyces palmae* CMU-AB204T	Multiple pathogens (e.g., *B. subtilis*, *E. coli*, *K. pneumoniae*, *S. aureus*, *P. aeruginosa*)	Gram-positive Gram-negative	Active compounds (AB204-A–F, anguinomycin, leptomycin) inhibit protein/DNA synthesis.	AB204-E/F is active against gram-positive bacteria; anguinomycin and leptomycin showed broad antimicrobial effects.	[148]
Palm Oil Mill Effluent	*S. aureus*, *E. coli*, *C. albicans*	Gram-positive Gram-negative Yeast	Disrupts cell walls or metabolic pathways (selective antimicrobial effect).	BDE-MA/PVA and BCX-MA/PVA hydrogels inhibited *S. aureus* (~31 mm); no effect on *E. coli* or *C. albicans.*	[149]
*Elaeis guineensis* Jacq leaves	*Staphylococcus aureus*	Gram-positive	Promotes wound healing via antimicrobial, epithelial, and collagen-enhancing properties.	Leaf extract eliminated *S. aureus* in rat wounds by day 16, similar to BETADINE^®^.	[150]

## 5. Advantages and Limitations of Palm Oil for Dental and Bone Tissue Engineering

Palm oil has garnered interest in dental and bone tissue engineering due to its abundant bioactive antioxidants, such as tocotrienols, tocopherols, and carotenoids, which are essential for reducing oxidative stress and promoting tissue regeneration [11]. The presence of these compounds, along with other antioxidants found in palm fruits and oils, contributes to antioxidant capacity, leading to a reduction in oxidative stress associated with chronic illnesses [151]. Apart from that, these substances have been demonstrated to protect fibroblasts and osteoblasts from oxidative damage, thereby promoting wound healing and bone development. Moreover, these palm oil-derived compounds provide fascinating opportunities for the production of biomaterials that serve as alternatives to synthetic or chemically based polymers.

Additionally, components derived from palm oil can be integrated into scaffolds, hydrogels, and coatings to improve their biocompatibility and oxidative stability, establishing them as potential opportunities for biomedical applications. It is emphasized that palm oil serves not only as a drug nanocarrier but also in the fabrication of biopolymeric scaffolding materials for medical applications in tissue and organ restoration and replacement [16]. Apart from that, palm oil also serves as a natural and sustainable resource, providing a cost-effective and biodegradable option for improving the bioactivity of tissue engineering scaffolds [152].

Besides, palm oil demonstrates antimicrobial and anti-inflammatory properties due to its high levels of bioactive compounds, including tocotrienols, carotenoids, and polyphenols. These compounds inhibit microbial growth and reduce inflammation, making palm oil a promising natural ingredient for wound healing, tissue regeneration, and infection control applications.

### 5.1. Potential Incorporation of Palm Oil into 3D Bioprinted Hydrogels for Oxidative Stress Modulation

A growing interest in the development of biocompatible and functionalized hydrogels for tissue engineering has prompted investigations into natural products exhibiting antioxidant properties. Palm oil has attracted interest due to its abundant bioactive compounds, which exhibit significant antioxidant and anti-inflammatory effects. These properties position palm oil as a viable candidate for integration into 3D bioprinted hydrogels, which are intended to modulate oxidative stress in the contexts of tissue regeneration.

Currently, there is a lack of research on the direct integration of palm oil within 3D bioprinted hydrogels. Nonetheless, the abundant antioxidant composition of palm oil, especially its tocotrienol and carotenoid constituents, positions it as a potential candidate for the modulation of oxidative stress in tissue regeneration. Although extensive studies have been conducted on the general uses of 3D printing in tissue engineering, the targeted integration of palm oil for the modulation of oxidative stress in bioprinted hydrogels has not been thoroughly investigated. The integration of 3D printing technology with antioxidant compounds presents considerable potential for the advancement of innovative therapeutic approaches. The integration of bioactive agents, including antioxidants or antimicrobial substances, into the hydrogel may significantly improve its therapeutic efficacy.

Recent studies have revealed that fractionated palm oil derivatives, including pressed palm fiber oil (PPFO), display notable antioxidant properties, primarily attributed to their elevated levels of water-soluble phenolics and various phytonutrients [153]. Antioxidant assays were performed on PPFO-based nanoemulgels, demonstrating that the bioactivity of these compounds was preserved following the formulation process. This indicated the capacity of palm oil to serve as a functional additive within biopolymeric systems, particularly in the context of modulating oxidative stress in regenerative applications. In addition, the antimicrobial activity of PPFO-based nanoemulgels was reported, with mechanisms that involve the disruption of bacterial peptidoglycan structures, consequently inhibiting bacterial growth. This enhances the therapeutic potential of palm oil derivatives, establishing them as versatile agents for application in tissue engineering scaffolds.

Apart from that, the antioxidant capabilities of hydrogels infused with oil palm leaf-derived total flavonoid-enriched extract (OPL-TFEE) for the reduction of free radicals have been investigated [154]. The results demonstrated that OPL-TFEE displayed notable antioxidant activity independently. The incorporation of OPL-TFEE into the hydrogel matrix preserved the antioxidant effect, resulting in results that were comparable to those observed with the extract alone. The findings indicate that incorporating OPL-TFEE into the hydrogels maintains their antioxidant properties and may function as a viable approach for targeted antioxidant delivery. The integration of bioactive compounds, including derivatives of palm oil, into hydrogel formulations significantly improves their therapeutic efficacy, providing a natural and effective approach to addressing oxidative stress.

Furthermore, the advancement of bioactive-loaded hydrogels for antioxidant delivery is significantly enhanced by the application of 3D bioprinting technology, which offers a unique approach to enhancing the antioxidant properties of these hydrogels. Moreover, 3D bioprinting technology has been recognized for its reproducibility and precise control over bioinks, which include essential cells and biomaterials. This precision extends to the scaffold’s structure and dimensions, allowing for functional tissue constructs tailored to individual patient needs [155]. This is achieved through the regulated distribution and release of active compounds, thereby improving their therapeutic efficacy [156]. Incorporating antioxidants into 3D printed matrices enables the achievement of controlled release and localized delivery, thereby addressing the limitations associated with traditional methods of antioxidant administration [157].

### 5.2. Impact of Palm Oil on Biocompatibility and Cellular Responses in Regenerative Medicine

Biocompatibility and non-toxicity are crucial properties for biomaterials used in medical and biomedical applications. Palm oil has garnered significant interest as a sustainable and bioactive element in the field of regenerative medicine. The incorporation of this element in biomaterials, particularly in the context of scaffold development, has demonstrated favorable outcomes for biocompatibility and cellular behavior. In dentistry, palm oil-derived polyols have been used and have exhibited multiple functional groups that facilitate polymer branching, resulting in an increased crosslinking density and the formation of three-dimensional interconnected structures. These characteristics contribute to enhancing mechanical strength and improving dimensional stability. The incorporation of palm oil-based polyol has the potential to enhance the biocompatibility of the synthesized composition [16]. This is mostly due to an abundance of natural antioxidants, which may prevent oxidative stress within the cellular environment.

Research on cellular responses has demonstrated that scaffolds made from palm oil can facilitate cell adhesion, proliferation, and viability. A 3-(4,5-dimethylthiazol-2-yl)-2,5-diphenyltetrazolium bromide (MTT) assay demonstrated that the palm fatty acid polyesters synthesized in this study exhibit high biocompatibility and are non-toxic to various cell types [51]. The finding is significant in that it implies that palm oil-based scaffolds can be used in medical applications without causing any adverse impacts on cellular activity. Furthermore, in vitro biocompatibility studies indicated that the polyurethane (PU) derived from palm oil films exhibited non-cytotoxic effects on human skin fibroblast cells [158].

Although palm oil is composed of numerous bioactive compounds that exhibit antioxidant and anti-inflammatory characteristics, including tocotrienols, tocopherols, and unsaturated fatty acids, it is crucial to note that crude or unprocessed palm oil must not be directly applied to cells in tissue culture or regenerative contexts. The hydrophobic characteristics, elevated lipid composition, and susceptibility to oxidation of palm oil may disrupt cell adhesion, nutrient transfer, and membrane stability, which could result in cytotoxic consequences.

### 5.3. Limitations

Despite its benefits, various limitations should be acknowledged when employing palm oil in dental and bone tissue engineering. A significant challenge is the bioavailability and stability of bioactive compounds in palm oil. Generally, palm oil contains hydrophobic antioxidants like vitamin E, ubiquinone, flavonoids, carotenoids, and retinoids that dissolve in fats. Vitamin E has two types, tocopherols and tocotrienols, which differ in their side chains [159,160,161]. Thus, the hydrophobic nature of these antioxidants may restrict their effective integration into aqueous-based biomaterials for tissue regeneration applications. Moreover, high concentrations of palm oil-derived compounds may present cytotoxic risks, requiring thorough dosage optimization to ensure biocompatibility.

Furthermore, challenges in processing and formulation, including phase separation and the semi-solid characteristics of palm oil, necessitate the use of advanced techniques such as nanoencapsulation to improve stability and enable controlled release in tissue engineering applications. Ethical and environmental issues associated with palm oil production necessitate consideration, highlighting the significance of employing sustainably sourced palm oil for biomedical applications. Innovative formulation strategies and additional research may facilitate the effective integration of palm oil into regenerative medicine and tissue engineering, addressing existing challenges.

## 6. Conclusions and Future Perspective

Palm oil and its derivatives demonstrate significant antimicrobial and antioxidant properties, positioning them as valuable options for biomedical, pharmaceutical, and food preservation applications. Bioactive compounds, including phenolics, flavonoids, and carotenoids, play a role in reducing oxidative stress and inhibiting microbial activity through mechanisms such as membrane disruption, enzyme inhibition, and modulation of ROS. The attributes of palm oil establish it as a sustainable and functional component in therapeutic formulations, wound dressings, and food packaging. Despite these advantages, numerous challenges need to be resolved prior to the widespread adoption of palm oil-based bioactive compounds in commercial applications. Standardizing extraction methods, optimizing bioavailability, and conducting thorough toxicological assessments are essential for ensuring efficacy and safety. The advancement of nanoencapsulation techniques and hybrid biomaterial formulations may improve the stability and controlled release of active compounds, thereby enhancing their therapeutic potential.

Future research must elucidate the molecular mechanisms underlying the bioactivities of palm oil, conduct extensive in vivo and clinical studies, and explore green processing technologies to enhance sustainability. Collaboration among researchers, industries, and policymakers can enhance innovation, ensuring that palm oil-derived antimicrobial and antioxidant applications significantly impact healthcare, biotechnology, and food security.

## Figures and Tables

**Table 1:** A comprehensive summary of synthesis techniques utilized for extracting and processing materials from the palm tree.

Raw Materials	Synthesis Technique	Synthesis Process	Outcome	References
Epoxidized palm olein (EPO)	Polycondensation	EPO was reacted with glutaric acid (1:0.7 molar ratio) at 210 °C for 6 h to produce polyols.	The resulting palm oil-based polyol is a liquid with acid value 1.95 mg KOH/g, hydroxyl value 84.50 mg KOH/g, molecular weight 6698, and viscosity decreasing from 24.55 Pa·s (25 °C) to 8.68 Pa·s (40 °C). Pour and cloud points: 12 °C.	[56]
Fresh oil palm leaves (OPLs)	Ultrasonic-aided extraction (UAE)	OPLs were dried, powdered, and extracted using ethanol (0–100%) and different solvent-to-solid ratios. Sonication (20 kHz, 130 W) preserved active compounds. Extracts were centrifuged, filtered, evaporated, freeze-dried, and stored at 4 °C.	Optimized UAE resulted in high extraction yield and rich phenolic content. Solvent concentration, time, and sonication intensity significantly influenced efficiency.	[64]
Fresh oil palm leaves (OPLs)	Ethanolic extraction	Leaves were dried, ground, and mixed with 50% ethanol. The mixture was sonicated (30 min), centrifuged, and freeze-dried. Extract was stored at 4 °C.	Moisture: 18.8%, ash: 5.2%, protein: 11.2%, fat: 7.1%, carbohydrates: 57.7%, energy: 339.5 kcal/100 g.	[74]
Fresh oil palm leaves (OPLs)	Maceration and reflux	Leaves were extracted using 70% ethanol. Extracts were dissolved in methanol to prepare a 1000 ppm stock solution.	Maceration yielded 14.93%, while reflux gave a higher yield of 27.26% due to enhanced extraction efficiency.	[65]
Freshly dumped shell of *Elaeis guineensis* Jacquin	Methanolic extraction	Shells were washed, dried, ground, and soaked in methanol for 72 h.	Extract contains tannins, alkaloids, terpenoids, saponins, phenolics, and flavonoids. Highest contents: phenolics (10.4%), tannins (5.67%), flavonoids (4.67%).	[75]
Palm fruit	Boiling and triturating extraction	Fruits were boiled, mashed, and mixed with water to separate the mesocarp. The mixture was filtered, concentrated, and refrigerated.	Extracts from ethyl acetate, dichloromethane, and n-hexane fractions contained high levels of fatty acids, especially hexadecanoic acid and oleic acid.	[71]
Palm leaves	Methanolic extraction	Tannins are extracted by boiling the leaves in methanol and filtering. Terpenes and alkaloids are obtained using an alkaline solution with dichloromethane, then separated and dried. Flavonoids are extracted by boiling in water, followed by liquid-liquid extraction with ethyl acetate and butanol.	Methanolic extract (ME): flavonoids, alkaloids, tannins, sterols, triterpenes. Tannin-free extract (ME-TF): flavonoids, alkaloids, terpenes. Terpene fraction (TF): only terpenes. Alkaloid fraction (AF): only alkaloids. Flavonoid fraction (FF): only flavonoids.	[76]
Fresh palm shells	Methanolic extraction	Shells were washed, dried, ground, and soaked in methanol for 72 h. The extract was filtered and concentrated.	Highest content: phenolics (11.4%), tannins (6.67%), and flavonoids (5.67%).	[72]
Palm fruit	Aqueous extraction	A total of 500 g of fruits were cleaned, boiled for 45 min, mashed, and filtered to separate the mesocarp. The extract was then filtered again, concentrated, and stored at 4 °C, yielding 95 g (19%).	Extraction content not reported. The yield was 19% (95 g).	[73]

**Table 2:** Comprehensive summary of studies on the antioxidant properties of palm oil.

Study Focus	Method of Identification	Antioxidant Components	Mechanism of Action (Antioxidant)	Antioxidant Findings	References
Phenolic content in crude vs. refined palm oils	DPPH Assay	Phenolic compounds.	Neutralize radicals, chelate metals.	Extra virgin olive oil (EVOO) (70%) > crude palm oil (CPO) (45%) > Crude Palm Kernel Oil (CPKO) (30%); refined oils lower; lowest IC50 in EVOO and CPO.	[110]
Lignin extracted from palm biomass	DPPH Assay	Phenolics, methoxyl, conjugated bonds.	Radical stabilization via resonance.	Lignin and its fractions (MeOH-F, ACT-F, and EtOH-F). MeOH-F and ACT-F had IC50 ~42–43 µg/mL; better than BHT and Irganox.	[113]
Lignin from mesocarp fibers with enzymes	Total phenolic content (TPC) and ferric reducing antioxidant power assay (FRAP)	Phenolics, carotenoids, tocopherols, tocotrienols.	Cell wall breakdown releases antioxidants.	Enzyme-treated oils had higher antioxidant activity; carotenoid yield increased by 153%.	[118]
Crude palm oil (CPO) and palm oil methyl ester (PME) antioxidant properties	DPPH Assay	Carotenoids, tocotrienols.	Scavenging radicals, lipid protection.	PME showed higher activity (69.3%) than CPO (30.1%); PME IC50 = 5.9 µg/mL.	[120]
Palm oil waste: antioxidant screening	DPPH Assay	Multiple phenolics.	Hydrogen donation, metal ion chelation.	Palm kernel cake had the highest phenolics and antioxidant capacity.	[79]
Palm oil from regions and nitric oxide (NO) scavenging	Nitric oxide (NO) scavenging activity assay	Phenolic (flavone): 7-dihydroxyflavone (chrysin).	Radical scavenging, Maillard reaction products.	Delta oil lowest (501.7 µg/mL); Ascorbic acid strongest (65.5 µg/mL).	[123]
nanostructured lipid carriers (NLC) to enhance β-carotene antioxidant activity	DPPH Assay ABTS Assay	Carotenoids, phenolics.	Encapsulation reduces oxidation and enhances activity.	βC-NLC exhibited significant antioxidant activity, achieving 91.47% in ABTS and 24.72% in DPPH free radical scavenging assays.	[124]
high-oleic palm oil (HOPO) based nanofibers	ABTS Assay	Carotenoids, phenolics.	Absorb/react with free radicals, preserve antioxidants.	Nanofibers had high activity and were stable after processing.	[125]
The protective role of red palm oil (RPO) in mitigating liver damage induced by lipopolysaccharide (LPS).	FRAP, ORAC, TEAC	Tocopherols, tocotrienols, β-/α-carotene.	Nrf2 activation, NF-κB inhibition, reduces oxidative stress.	RPO restored FRAP; no change in ORAC/TEAC values.	[126]
ultra-high pressure (UHP)-treated palm fruits	DPPH, FRAP, ABTS	Phenolic acids, flavonoids.	Electron/hydrogen donation, ROS reduction, metal chelation.	UHP improved antioxidant levels; strong correlation with total phenolic content.	[127]

## Data Availability

Not applicable.

## Abbreviations

The following abbreviations are used in this manuscript:

AP-1	Activator Protein 1
CPO	Crude Palm Oil
DPPH	2,2-Diphenyl-1-Picrylhydrazyl
EPO	Epoxidized Palm Olein
ERK	Extracellular Signal-Regulated Kinase
FGF	Fibroblast Growth Factor
JNK	c-Jun N-Terminal Kinase
MAPK	Mitogen-Activated Protein Kinase
Nf-κB	Nuclear Factor Kappa-Light-Chain-Enhancer of Activated B Cell
NLC	Nanostructured Lipid Carrier
Nrf2	Nuclear Factor Erythroid 2-Related Factor 2
OPG	Osteoprotegerin
OPL	Oil Palm Leaf
PKO	Palm Kernel Oil
PME	Palm Methyl Ester
PPP	Palm-Based Polyester Polyol
RANKL	Receptor Activator of Nuclear Factor Kappa-Β Ligand
ROS	Reactive Oxygen Species
RPO	Red Palm Oil
SL	Sophorolipid
